# Solution-Processed Metal Oxide Nanostructures for Carrier Transport

**DOI:** 10.3390/nano13081331

**Published:** 2023-04-11

**Authors:** Sheng-Hsiung Yang

**Affiliations:** Institute of Lighting and Energy Photonics, College of Photonics, National Yang Ming Chiao Tung University, Tainan 711010, Taiwan; yangsh@nycu.edu.tw

Metal oxide semiconductors represent a unique class of materials that show prominent optoelectronic applications nowadays. Typical p-type metal oxides, such as vanadium oxide (VO_x_), nickel oxide (NiO_x_), and cobalt oxide (CoO_x_), have the advantages of high hole mobility, superior stability, and solution processibility. On the other hand, several representative n-type metal oxide materials, including zinc oxide (ZnO), titanium dioxide (TiO_2_), and tin dioxide (SnO_2_), show benefits in device performance due to their high electron mobility, long-term stability, solution-processing capability, and relatively high abundance on earth. Apart from thin films, nanostructured metal oxides have also been developed to increase the active surface area as well as response to incident energy, which is especially useful for carrier transport in miscellaneous optoelectronic devices. Different kinds of nanostructures, including nanoparticles (NPs), nanowires, nanorods, nanotubes, nanosheets, nanowalls (NWLs), and nanoflowers, are achievable via the solution process to reduce production cost and to expand research diversity. This Special Issue presents six research articles that demonstrate the preparation of metal oxide nanostructures via the solution process for different applications, including hydrogen production [1,2], perovskite solar cells (PSCs) [3,4], quantum dot light-emitting diodes (QLEDs) [5], and organic light-emitting diodes (OLEDs) [6].

The hydrothermal method is usually adopted to produce nanostructured metal oxides. Jiang and Yang prepared nickel (Ni)-doped ZnO NWLs with different pore sizes and layer roughness via the hydrothermal method [1]. They found that the pore size of Ni-doped ZnO NWLs could be adjusted by changing the concentration of the precursor hexamethylenetetramine (HMT) from 1 to 5 mM. As the HMT concentration increased, the pores became larger with increasing surface roughness. The electrical conductivity of the ZnO NWLs was enhanced by Ni doping, whereas it decreased with increasing HMT concentration, revealing that Ni doping and HMT concentration adjustment were the two key approaches to control the morphology and electrical properties of ZnO NWLs. The results showed that the Ni-doped ZnO NWLs using 1 mM HMT had the highest electrochemical behavior through water splitting. Tsai and Su reported the synthesis of perovskite LaFeO_3_ doped with varying concentrations of Ni to form double perovskite LaFe_1−x_Ni_x_O_3_ [2]. The gel solution of LaFe_1−x_Ni_x_O_3_ was then deposited on the substrate by spin coating, followed by decoration of sea urchin-like gold (Au) NPs using electrophoresis to enhance the surface plasmon resonance effect. The Au-coated LaFe_1−x_Ni_x_O_3_ is an n-type semiconductor with a bandgap larger than 1.23 eV (H^+^/H_2_). With the decoration of Au NPs, the coupling effect between the LaFe_1−x_Ni_x_O_3_ and Au NPs and the more active sites from the Au NPs improved the hydrogen efficiency. The maximum real hydrogen production of the Au NP-coated LaFe_1−x_Ni_x_O_3_ was up to 43,800 μmol g^−1^ h^−1^ in ethanol.

Nanostructured metal oxides can serve as the carrier transport layer in regular or inverted PSCs, depending on their charge transport capability. Yelzhanova et al. prepared different SnO_2_ nanostructures via a solvothermal method by controlling several growth parameters [3], including substrate orientation, types of seed layer, amounts of acetic acid, deionized water-to-ethanol ratios, and growth pressure and time. The results revealed that SnO_2_ nanorods tended to grow in a single direction on the seed layer that was perpendicular to the substrate. SnO_2_ nanorod arrays with reasonable free space among the nanostructures could be utilized as the electron transport layer to accommodate perovskite grains to construct regular PSCs. The optimized PSC exhibited an open-circuit voltage (V_OC_) of 1.11 V, a short-circuit current density (J_SC_) of 22.9 mA/cm^2^, a fill factor (FF) of 0.65, and a power conversion efficiency (PCE) of 16.6% with oxygen plasma treatment of the SnO_2_ nanostructures prior to the deposition of the perovskite absorbing layer. Chang et al. developed a three-stage method to prepare NiO_x_ nanoflakes [4]. The three-stage method is as follows: (1) formation of ZnO nanorods by a hydrothermal synthesis, (2) chemical bath deposition of mesoporous NiO_x_ on ZnO nanorods, and (3) ZnO removal by chemical etching. The formed NiO_x_ nanoflakes helped to grow perovskite nanocrystals with larger grain sizes and fewer grain boundaries when compared to the NiO_x_ film. Due to the hole transporting nature of NiO_x_, inverted PSCs based on the NiO_x_ nanoflakes and the NiO_x_ thin film as the hole transport layer were fabricated and evaluated. The optimal PSC using the NiO_x_ nanoflakes showed a V_OC_ of 0.99 V, a J_SC_ of 20.5 mA/cm^2^, a FF of 70%, and a PCE of 14.21%, which was significantly better than the controlled device based on the NiO_x_ thin film. 

In addition to photovoltaic devices, nanostructured metal oxides can be utilized in the light-emitting field. Lee et al. synthesized ZnO quantum dots capped with oleic acid (OA) ligands to decrease the work function of 3.15 eV for ZnO to 2.72–3.08 eV for ZnO-OA [5], while the valence band was increased up to 7.22–7.23 eV. By using ZnO-OA/ZnO double ETLs with matched energy level alignment, the optimized CdSe QLED showed 5–9% enhancement in maximum luminance and 16–35% improvement in current efficiency when compared to the controlled device using single ZnO ETL. Kang et al. incorporated ZnO NPs as the electron-injection layer in deep-blue OLEDs [6], using two new molecules, 2-(9H-carbazol-9-yl)-5-(2,12-di-tert-butyl-5,9-dioxa-13b-boranaphtho[3,2,1-de]anthracen-7-yl)-5H-benzo[b]carbazole (TDBA-BCZ) and 5-(2,12-di-tert-butyl-5,9-dioxa- 13b-boranaphtho[3,2,1-de]anthracen-7-yl)-8-phenyl-5,8-dihydroindolo[2,3-c] carbazole (TDBA-PCZ), as the emitters. The ZnO NP layer composed of dense spherical particles was obtained by spin coating from a commercial ZnO NP solution. The best OLED based on TDBA-BCZ exhibited a turn-on voltage (V_on_) of 4.89 V, a maximum current efficiency of 8.67 cd/A, an external quantum efficiency (EQE) of 7.73%, and a maximum electroluminescence (EL) wavelength at 428 nm (deep-blue region). The optimized OLED based on TDBA-PCZ showed a V_on_ of 4.29 V, a maximum current efficiency of 14.24 cd/A, an EQE of 10.58%, and a maximum EL at 461 nm (blue region). The CIE’1931 coordinates for the TDBA-BCZ and TDBA-PCZ devices were (0.161, 0.046) and (0.151, 0.155), respectively. These results reveal the potential use of solution-processed blue OLEDs for display applications.

In summary, this Special Issue includes a compilation of articles that demonstrate recent developments of nanostructured metal oxide materials by the solution process and their applications in various optoelectronic fields. It showcases the contributions of the presented articles and provides insights into recent developments; thus, we highly recommend this Special Issue for the academic and industrial communities to read and to further contribute to this research topic.
